# Screening for Mutations in the *TBX1* Gene on Chromosome 22q11.2 in Schizophrenia

**DOI:** 10.3390/genes7110102

**Published:** 2016-11-22

**Authors:** Lieh-Yung Ping, Yang-An Chuang, Shih-Hsin Hsu, Hsin-Yao Tsai, Min-Chih Cheng

**Affiliations:** 1 Department of Psychiatry, Yuli Branch, Taipei Veterans General Hospital, Hualien 98142, Taiwan; minipyng@gmail.com (L.-Y.P.); yc2579@gmail.com (Y.-A.C.); filvhsu@gmail.com (S.-H.H.); ashleytsai0808@gmail.com (H.-Y.T.); 2 Center for General Education, St. Mary’s Junior College of Medicine, Nursing and Management, Yilan County 26644, Taiwan

**Keywords:** schizophrenia, TBX1, rare mutation, 22q11.2 deletion

## Abstract

A higher-than-expected frequency of schizophrenia in patients with 22q11.2 deletion syndrome suggests that chromosome 22q11.2 harbors the responsive genes related to the pathophysiology of schizophrenia. The *TBX1* gene, which maps to the region on chromosome 22q11.2, plays a vital role in neuronal functions. Haploinsufficiency of the *TBX1* gene is associated with schizophrenia endophenotype. This study aimed to investigate whether the *TBX1* gene is associated with schizophrenia. We searched for mutations in the *TBX1* gene in 652 patients with schizophrenia and 567 control subjects using a re-sequencing method and conducted a reporter gene assay. We identified six SNPs and 25 rare mutations with no association with schizophrenia from Taiwan. Notably, we identified two rare schizophrenia-specific mutations (c.-123G>C and c.-11delC) located at 5′ UTR of the *TBX1* gene. The reporter gene assay showed that c.-123C significantly decreased promoter activity, while c.-11delC increased promoter activity compared with the wild-type. Our findings suggest that the *TBX1* gene is unlikely a major susceptible gene for schizophrenia in an ethnic Chinese population for Taiwan, but a few rare mutations in the *TBX1* gene may contribute to the pathogenesis of schizophrenia in some patients.

## 1. Introduction

Schizophrenia is a severe chronic mental illness characterized by abnormal perceptions, thought disturbances, bizarre behaviors, and impaired cognitive function [1]. This disease affects approximately 1% of the general population worldwide. The etiology and pathogenesis of schizophrenia are still unclear today. Many researchers have found a higher-than-expected frequency of 22q11.2 deletions in patients with schizophrenia, suggesting that chromosome 22q11.2 harbors the responsive genes for the pathophysiology of schizophrenia [2,3,4].

22q11.2 deletion syndrome (22q11.2DS), also known as DiGeorge syndrome or velocardiofacial syndrome, is a disease caused by an interstitial microdeletion on chromosome 22 with an incidence between 1:4000 and 1:6000 live birth [5,6]. The clinical characteristic of 22q11.2DS, which is complex and variable, include craniofacial and cardiovascular anomalies, immunodeficiency, short stature, and hypocalcemia [6]. There is substantial epidemiological evidence that 22q11.2DS is characterized by a greatly increased risk for schizophrenia [7,8,9,10]. Moreover, genomic evaluation of copy number variation has established that 22q11.2 deletion of strong effect increases risk for schizophrenia [4,11,12]. These studies have highlighted that the 22q11.2 region harbors genes causally implicated in schizophrenia in a subset of patients.

The *TBX1* gene, encoding a member of the transcription factors that share a common DNA-binding domain known as the T-box [13,14], lies within the 22q11.2 region and is a major candidate gene for 22q11.2DS [14,15,16,17,18]. The *TBX1* gene was conserved across several species and was expressed in the human brain [19,20]. Mutations and haploinsufficiency of the *TBX1* gene are sufficient to cause reduced prepulse inhibition, a behavioral abnormality that is associated with schizophrenia endophenotype [21,22]. Loss of the *TBX1* gene disrupts cortical development [23] and global brain vascular defect [24] in mice. Congenic *TBX1* heterozygous mice displayed the autism-related behavioral phenotypes [25,26]. There are overlapping symptoms between autism and schizophrenia, which may suggest that these two diseases share some common biological basis in their pathogenesis [27]. Therefore, it is plausible that the deletion or disruption of the *TBX1* gene may alter the expression of genes required for proper development and function of neuronal circuits in the central nerve system, and eventually lead to the formation of schizophrenia.

It is assumed that the genetic underpinning of schizophrenia can be attributed to the juncture of multiple common variants with low penetrance [28,29]. Conversely, there is an increasing appreciation that schizophrenia can be associated with rare mutations with high clinical penetrance in some patients [30,31]. Here, we aimed to examine whether there are common or rare genetic variants of the *TBX1* gene associated with schizophrenia. To test this possibility, we systemically searched for genetic variants in all the exons of the *TBX1* gene in a sample of patients with schizophrenia and control subjects from Taiwan and conducted a reporter gene activity assay to characterize genetic variants located at 5′ UTR of the *TBX1* gene.

## 2. Materials and Methods

### 2.1. Subjects

Patients fulfilling the diagnostic criteria of schizophrenia defined by the four version of the Diagnostic and Statistical Manual of Mental Disorders were recruited into this study. The diagnosis of schizophrenia was based on a clinical interview and review of medical records by senior psychiatrists with consensus. Exclusion criteria include psychosis due to general medical condition, substance-related psychosis, and mood disorder with psychotic features. Control subjects were recruited from a medical center’s Department of Family Medicine located in Eastern Taiwan. All subjects were Han Chinese from Taiwan. The study was approved by the Institution Review Board, and written informed consent was obtained after the procedures were fully explained. We recruited 652 patients with schizophrenia and 567 age- and sex-matched non-psychotic subjects as the control. Genomic DNA was prepared from peripheral blood cells according to standard protocols.

### 2.2. Nomenclature and Reference Sequences

We acquired the gene information of the *TBX1* gene from NCBI (http://www.nhri.nlm.nih.gov) and UCSC database (http://genome.ucsc.edu/index.html). The nomenclature of these sequence variations follows the “Nomenclature for description of human sequence variations” [32]. The GenBank accession numbers of the reference sequences for the three isoforms of the *TBX1* gene used in this study are NM_080646.1, NM_005992.1, and NM_080647.1

### 2.3. PCR-Based Sequencing 

Optimal PCR primer sequences were generated to each exon of the *TBX1* gene using Primer3 website (http://bioinfo.ut.ee/primer3-0.4.0/primer3/). Primer sequences, optimal annealing temperatures, and size of each amplicon are available on request. Genomic DNA (75 ng) was amplified in a reaction volume of 15 µL containing 0.5 µM each of forward and reverse primer, 0.2 mM of dNTP, 50 mM of KCl, 1.5 mM of MgCl_2_, 0.1% vol/vol of Triton X-100, 10 mM of Tris-HCl (pH 9.0 at 25 °C), and 2.5 U PowerTAQ DNA polymerase (GeneTek BioScience Inc., Taipei, Taiwan). PCR cycling conditions consisted of an initial denaturation at 95 °C for 1 min, the optimal annealing temperature of each amplicon for 1 min, and 72 °C for 1 min. After PCR amplification, aliquots of PCR products were processed using an Illustra^TM^ ExoProStar^TM^ 1-Step Kit (GE Healthcare Bio-Sciences Corp., NJ, USA) to remove residual primers and dNTPs following the manufacturer’s protocol. The purified PCR products were sequencing using an ABI Prism™ BigDye™ Terminator Cycle Sequencing Ready Reaction Kit (version 3.1) and an ABI autosequencer 3730 (Perkin Elmer Applied Biosystem, Foster City, CA, USA) according to the manufacturer’s protocol. Repeat PCR and sequencing verified all variants in both directions.

### 2.4. In Silico Analysis

The potential functional consequences of missense mutations were predicted using the PolyPhen-2 (http://genetics.bwh.harvard.edu/pph2/), the PMut (http://mmb.pcb.ub.es/PMut/), and SIFT (http://sift.jcvi.org/). The alteration of putative transcription factor binding sites by the 5′ UTR variants of the *TBX1* gene was evaluated using PROMO (http://alggen.lsi.upc.es/cgi-bin/promo_v3/promo/promoinit.cgi?dirDB=TF_8.3).

### 2.5. Reporter Gene Activity Assay

Genomic DNA from the subjects were used for constructing the inserts for the reporter gene activity assay. For functional characterization of c.-123C, c.-120T, sense primer (5′-CAGACCCTGCGACCCCTA-3′) and antisense primer (5′-AGTGTTCCTCCCTCCTCAC-3′) were used to obtain amplicon containing identified genetic variants. For functional characterization of c.-84A, c.-11delC, sense primer (5′-GTTCAGCATCGCCTCTCTG-3′) and antisense primer (5′-CAAGAGCTGCCTCCACCTAC-3′) were used to obtain amplicon containing identified genetic variants. The PCR fragments were cloned into pCR-II-TOPO vector (Invitrogen, Carlsbad, CA, USA) then subcloned into the pGL3-basic vector (Promega, Madison, WI, USA) using HindIII and XhoI recognition sites, and the authenticity of each construct was verified by sequencing. SK-N-SH neuroblastoma cells were cultured in 96-well plates at 3000 cells per well in MEM supplemented with 1 mM sodium pyruvate, 0.1 mM non-essential amino acids, penicillin-streptomycin (Invitrogen, Carlsbad, CA, USA), and 10% fetal bovine serum. The cells were cotransfected with 200 ng of reporter plasmid and 10 ng of pRL-TK (Promega, Madison, WI, USA) as an internal control reporter using 0.5 µL of the Lipofectamine^TM^ 2000 (Invitrogen, Carlsbad, CA, USA), and six replicates were performed for each treatment. At 30 h after transfection, cells were lysed and the luciferase activities were measured using the Dual-Luciferase Reporter Assay System according to the manufacturer’s instructions (Promega, Madison, WI, USA). The firefly luciferase activity was normalized against the Renilla luciferase activity in each transfection.

### 2.6. Statistical Analysis

Deviation from the Hardy–Weinberg equilibrium of the genotype distribution in both the patient and control groups was examined with the chi-square test. Differences in allele, genotype, and estimated haplotype frequencies between patients and controls were evaluated using an online computer platform SHEsis (http://analysis.bio-x.cn/myAnalysis.php). A *p*-value of less than 0.05 was considered statistically significant. The differences in the frequency of rare mutations between patient and control groups were assessed using Fisher’s exact test with a significance level at 0.05 (two-tailed).

## 3. Results

### 3.1. Genetic Analysis of the TBX1 Gene

**Figure 1:**
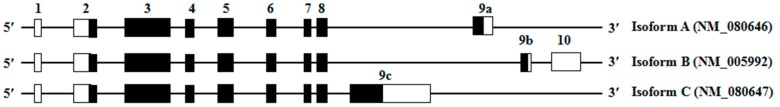
The schematic genomic structure of the *TBX1* gene and locations of molecular variants analyzed in this study. The black box indicates the protein-coding region; the white box indicates the untranslated region.

The human *TBX1* gene has three isoforms that share exons 1–8 but differ in the terminal exons 9A, 9B/10, and 9C and comprises twelve exons that span approximately 27 kb on chromosome 22q11.21. The genomic structure of the *TBX1* gene is illustrated in Figure 1. We identified a total of 31 genetic variants of the *TBX1* gene in patients with schizophrenia and control subjects, including six common SNPs (rs737868, rs41298814, rs2301558, rs72646967, rs4819522, and rs5746826) with minor allele frequency (MAF) above 5% and 25 rare mutations with MAF below 5%. Six common SNPs were selected for genetic association analysis. The genotype and allele frequencies of these six SNPs are listed in Table 1. There is no significant deviation of these common SNPs from the Hardy–Weinberg equilibrium in either patient or control group except for rs72646967. However, there are no significant differences in the genotype or allele frequency of these common SNPs between patients with schizophrenia and control subjects (Table 1).

Twenty-five rare variants include 7 missense variants (p.Asp151Glu, p.Asp155Asn, p.Glu257Ala, p.Arg342Gln, pVal359Ala, p.Ala393Thr, and p.Arg396His) and 13 variants (c.-123G>C, c.-120G>T, c.-84G>A, c.-11delC, c.*13C>T, c.*108G>C, c.*165_166insG, c.*315C>T, c.*399G>A, c.*398-406delTGTAGATAC, c.*24C>A, c.*123G>A, and c.*170C>T) located at the untranslated region (Table 2). Among these 25 rare variants, 11 (c.-123G>C, c.-11delC, p.Asp151Glu, p.Glu257Ala, p.Arg342Gln, pAla353= , p.Pro398=, c.*315C>T, p.Arg396His, c.*123G>A, and c.*170C>T) were detected in schizophrenic patients only, while 9 (c.-120G>T, c.-84G>A, p.Asp155Asn, p.Ala256=, p.Thr271=, c.*13C>T, c.*108G>C, c.*399G>A, and p.Val359Ala) were detected in control subjects only. Five mutations (c.*164_*165insG, c.*398-406delTGTAGATAC, p.Ala393Thr, p.Asp395=, and c.*24C>A) were detected in both patients and control subjects. There is no increasing burden of these rare variants in the patient group as compared to the control group (39/652 cases versus 36/567 controls, *p* = 0.80). The functional impact of the missense mutations was assessed by the amino acid analysis programs Polyphen-2, PMut, and SIFT to identify those deemed to be possibly or probably deleterious to protein function (Table 2). The bioinformatic analysis predicts that the c.-123G>C may disrupt transcription factor binding sites of AP-2 and LVc, and the c.-120G>T may disrupt transcription factor binding sites of AP-2 only.

### 3.2. Reporter Gene Activity Assay

We performed a reporter gene activity assay to assess the potential regulatory impact of four variants (c.-123G>C, c.-120G>T, c.-84G>A, and c.-11delC) on the expression of the *TBX1* gene. The mutant c.-123G>C appeared to significantly decrease promoter activity compared with the wild type in the SK-N-SH cells, while two mutants (c.-84G>A and c.-11delC) appeared to significantly increase promoter activity (Table 3).

### 3.3. Clinical Findings of the Patients with Rare Mutations

The patient carrying the c.-123G>C mutation was a 50-year-old lady who has suffered from paranoid schizophrenia since the age of 20. Her mother was also a victim of schizophrenia; otherwise, no other family members (including five other siblings) had a diagnosis of mental illness. She was born at full term, and the developmental history was unremarkable. After she graduated from college, she gradually developed auditory-hallucination disturbance, derailed speech, the delusion of self-reference, and deterioration of occupational and social functions. She denied any history of illicit drugs or alcohol misuse, head injury, central nervous system (CNS) infection, or epilepsy. Although she responded well to the antipsychotic medication, she only showed fair drug adherence such that her psychotic symptoms occasionally relapsed. As a result, she was hospitalized to psychiatric wards several times. Both her social and occupational functions deteriorated progressively throughout the years; currently, she is admitted to a chronic nursing unit with the existence of residual psychotic symptoms.

The patient carrying the c.-11delC mutation was a 51-year-old woman who has been diagnosed with schizophrenia since the age of 20. One of his paternal aunts was also a victim of psychosis, but the diagnosis was uncertain. Both her birth and developmental history were insignificant. At present, she occasionally showed mood irritability, auditory-hallucination disturbance, tangentiality in speech, and the ideation of persecution, which moderately impaired her occupational and social functions. She denied any history of illicit drugs or alcohol misuse, head injury, CNS infection, or epilepsy. Both her social and vocational functions deteriorated progressively throughout the years; currently, she is admitted to a chronic nursing unit with the existence of residual psychotic symptoms.

## 4. Discussion

In this study, we resequenced the *TBX1* gene in patients with schizophrenia and control subjects from Taiwan and discovered six common SNPs, and further analysis showed no association of the SNPs with schizophrenia, in line with results from the other genetic association studies [33,34].

In addition to common SNPs, we identified 25 rare mutations of the *TBX1* gene in this sample. However, no increasing burden of rare mutations was found in the patient group, suggesting rare mutations of the *TBX1* gene occurred equally in both patients with schizophrenia and control groups. However, Paylor et al. identified a 23 bp frameshift deletion of the *TBX1* gene, which may disrupt the central domain of a highly conserved nuclear localization signal of the wild-type TBX1 protein from one family member with Asperger syndrome [21]. A report links mutation in the *TBX1* gene to developmental delay [35]. In our study, we identified two schizophrenia-specific variants (c.-123G>C and c.-11delC) with abnormal promoter activity, suggesting that abnormal *TBX1* gene expression may contribute to the pathogenesis of schizophrenia in some patients. Taken together, these studies suggest that the *TBX1* gene is likely a common susceptible gene among several mental disorders such as schizophrenia, autism, and mental retardation.

In sillico analysis predicts that the mutant c.-123G>C disrupts transcription factor binding sites of AP-2 and LVc. We suggested that a possible regulatory function of pathological mutations in 5′ UTR of the *TBX1* gene modulates gene expression via trans-acting genetic modifiers. In addition, we identified two schizophrenia-specific missense mutations (p.Asp151Glu and p.Glu257Ala) located within the T-box domain. The T-box domain, which is highly conserved in all T-box proteins, is responsible for DNA binding and is likely essential for dimerization of the TBX1 protein. We assumed that these two missense mutations are likely to impair DNA binding activity of the TBX1 protein, but further functional analyses are needed to verify our speculation. As some of the mutations in the control group were predicted to have a damaging effect on the TBX protein, it is likely that the penetrance and clinical manifestation of mutations might be modified by other genetic or environmental factors [3,36].

This study had the following limitations. Firstly, the effect of all of the reported mutations on TBX1 protein function is still unknown, and the identification of functional domains is an important subject for future research. Secondly, schizophrenia is a heterogeneity disorder, and additional risk factors and other genes in the chromosome 22q11.2 deleted region may also contribute to illness progression. Lastly, we also found several mutations in the control group owing to the incomplete penetrance of the 22q11.2DS phenotypes. Thus, the finding of a normal carrier does not necessarily exclude a disease-causative role for the mutation.

## 5. Conclusions

This study suggests that the common genetic variants of the *TBX1* gene may not play a major role in conferring susceptibility to schizophrenia. Nevertheless, some rare mutations in the *TBX1* gene with a possible damaging effect may be present in some patients.

## Figures and Tables

**Table 1:** Genotype and allele frequencies of molecular variants of the *TBX1* gene in patients with schizophrenia (SZ) and control subjects (Ctrl).

Variant	Group	n	Genotype			HWE	*p*	Allele		*p*
c.-85G>C (rs737868)			G/G	G/C	C/C		0.60	G	C	0.46
	SZ	564	105(18.6%)	269(47.7%)	190(33.7%)	0.57		479(42.5%)	649(57.5%)	
	Ctrl	546	89(16.3%)	269(49.3%)	188(34.4%)	0.66		447(40.9%)	645(59.1%)	
p.Phe140= (rs41298814)			T/T	T/C	C/C		0.40	T	C	0.20
	SZ	594	161(27.1%)	293(49.3%)	140(23.6%)	0.77		615(51.8%)	573(48.2%)	
	Ctrl	567	166(29.3%)	285(50.3%)	116(20.4%)	0.75		617(54.4%)	517(45.6%)	
p.Leu222= (rs2301558)			C/C	C/T	T/T		0.48	C	T	0.77
	SZ	518	397(76.6%)	114(22.0%)	7(1.4%)	0.71		908(87.6%)	128(12.4%)	
	Ctrl	536	419(78.2%)	106(19.8%)	11(2.0%)	0.17		944(88.1%)	128(11.9%)	
p.Asn397His (rs72646967)			A/A	A/C	C/C		0.20	A	C	0.07
	SZ	529	151(28.5%)	238(45.0%)	140(26.5%)	0.02		540(51.0%)	518(49.0%)	
	Ctrl	524	167(31.9%)	242(46.2%)	115(22.0%)	0.12		476(55.0%)	472(45.0%)	
p.Thr350Met (rs4819522)			C/C	C/T	T/T		0.78	C	T	0.64
	SZ	653	502(76.9%)	138(21.1%)	13(0.2%)	0.34		1142(87.4%)	164(12.6%)	
	Ctrl	545	423(77.6%)	114(20.9%)	8(1.5%)	0.92		960(88.1%)	130(11.9%)	
c.*121G>T (rs5746826)			G/G	G/T	T/T		0.38	G	T	0.16
	SZ	650	110(16.9%)	306(47.1%)	234(36.0%)	0.56		526(40.5%)	774(59.5%)	
	Ctrl	544	107(19.7%)	257(47.2%)	180(33.1%)	0.38		471(43.3%)	617(56.7%)	

HWE = Hardy–Weinberg equilibrium.

**Table 2:** Distributions and bioinformatics analyses of rare mutations in the *TBX1* gene identified in this study.

Variant (*TBX1* Isoform)	RS Number	Frequency		Amino Acid Change	In Silico Analysis			
		SZ	Ctrl		Transcription Factor ^a^	Pmut	PolyPhen-2	SIFT
c.-123G>C (isoform C)	None	1/638	0/534	N/A	Wild-type: AP-2, LVc	N/A	N/A	N/A
c.-120G>T (isoform C)	None	0/637	1/534	N/A	Wild-type: AP-2	N/A	N/A	N/A
c.-84G>A (isoform C)	None	0/564	1/546	N/A	N/A	N/A	N/A	N/A
c.-11delC (isoform C)	rs78833362	1/565	0/546	N/A	N/A	N/A	N/A	N/A
c.453T>A (isoform C)	rs778041960	1/594	0/567	p.Asp151Glu	N/A	Neutral	Benign	Deleterious
c.463G>A (isoform C)	rs374011293	0/594	3/567	p.Asp155Asn	N/A	Neutral	Possibly damaging	Deleterious
c.768C>T (isoform C)	rs759225333	0/553	1/548	p.Ala256=	N/A		N/A	N/A
c.770A>C (isoform C)	None	1/553	0/548	p.Glu257Ala	N/A	Neutral	Benign	Deleterious
c.813C>T (isoform C)	rs61730282	0/449	1/548	p.Thr271=	N/A		N/A	N/A
c.1025G>A (isoform C)	rs549715785	2/535	0/515	p.Arg342Gln	N/A	Pathological	Benign	Deleterious
c.1059A>G (isoform C)	rs13054377	1/426	0/528	pAla353=	N/A	N/A	N/A	N/A
c.1194C>A (isoform C)	None	1/531	0/400	p.Pro398=	N/A	N/A	N/A	N/A
c.*13C>T (isoform C)	rs543378212	0/489	2/525	N/A	N/A	N/A	N/A	N/A
c.*108G>C (isoform C)	None	0/500	1/525	N/A	N/A	N/A	N/A	N/A
c.*164_*165insG (isoform C)	rs41298842	10/500	10/534	N/A	N/A	N/A	N/A	N/A
c.*315C>T (isoform C)	None	1/488	0/535	N/A	N/A	N/A	N/A	N/A
c.*399G>A (isoform C)	None	0/487	3/534	N/A	N/A	N/A	N/A	N/A
c.*398-406 delTGTAGATAC (isoform C)	None	1/488	1/534	N/A	N/A	N/A	N/A	N/A
c.1076T>C (isoform A)	rs200361367	0/543	1/544	p.Val359Ala	N/A	Pathological	Possibly damaging	Tolerated
c.1177G>A (isoform A)	None	1/652	1/544	p.Ala393Thr	N/A	Neutral	Benign	Deleterious
c.1185C>T (isoform A)	None	3/652	1/544	p.Asp395=	N/A	N/A	N/A	N/A
c.1187G>A (isoform A)	rs207477905	1/542	0/544	p.Arg396His	N/A	Neutral	Benign	Deleterious
c.*24C>A (isoform A)	rs41298008	12/542	9/544	N/A	N/A	N/A	N/A	N/A
c.*123G>A (isoform A)	None	1/650	0/544	N/A	N/A	N/A	N/A	N/A
c.*170C>T (isoform B)	None	1/588	0/326	N/A	N/A	N/A	N/A	N/A

**^a^** Transcription factors predicted by PROMO; SZ = schizophrenia; Ctrl = control; N/A = not available.

**Table 3:** Reporter gene activity assay of rare mutations the *TBX1* gene.

Mutation	Fluc/Rluc (n = 6)	*p* Value
Wild-type	36.96 ± 3.92	
c.-123C	18.19 ± 2.39	<0.01 *
c.-120T	36.17 ± 3.64	=0.72
pGL3-Enhancer	12.83 ± 2.00	
Wild-type	18.02 ± 1.70	
c.-84A	43.90 ± 4.21	<0.01 *
c.-11delC	52.39 ± 8.76	<0.01 *
pGL3-Enhancer	18.50 ± 3.73	

Fluc/Rluc indicates the luciferase activity normalized by Renilla activity; *p*-value shows the significance of the difference between mutant and wild type (two-tailed *t*-test). * *p* < 0.05.
